# The International Homœopathic Congress of Paris

**Published:** 1890-04

**Authors:** 


### THE INTERNATIONAL HOMŒOPATHIC CONGRESS OF PARIS.

Translated from the French by Walter M. James, M. D.

The late International Homœopathic Congress of Paris held its sessions in the Trocadero the 21st, 22d, and 23d of August last. The following is the list of officers:

President, Dr. Jousset; Vice-Presidents, Drs. Lêon Simón, Sr.; Richard Hughes, of Brighton, England; Gailliard, of Brussels, Belgium; General Sec. and Treas., Dr. Mark Jousset. Assistant Secretaries, Drs. Parenteau and Vincent Léon Simón.

Allow us to say before all, in view of the number and class of its members, and the importance of the subjects discussed, the late Homœopathic Congress is one of the most remarkable ever held in Europe. Active and honorary members together, we were more than one hundred attendants, one-half of whom were foreigners. It can, therefore, be said it was the most international meeting we have attended up to this date. The following is a list of members present not Parisians :

*France.*—Messrs. Bernay, Gallavardin, Imbert de la Touche, of Lyons ; Chapiel, of Bordeaux ; Conqueret, of Versailles ; De Créquy, of Amiens ; Daniel, of Marseilles ; Garcin, of Aix ; Malapert du Peux, of Lille ; Pellerin, of Algeria ; Gras, of St. Nazaire.

*Germany.*—Elb, of Dresden ; Alexander von Villers, of Dresden ; Grünewald, of Frankfort ; Lutz, of Koethen ; Gailliard, of Brussels.

*Bulgaria.*—Mircowitz, of Slivno.

*Spain.*—Pellicer, Jr., and Alexander Soler, of Madrid ; Juan Sanllehy and Sabater, of Barcelona.

*Greece.*—M. Psilla, of Patras.

*England.*—R. Hughes, of Brighton ; Drysdale, of Liverpool ; Dudgeon, of London ; Suss Hahnemann, Roth, and Maurice, of London.

*Italy.*—Alleori and Vincenzo Liberali, of Rome ; Baldelli, of Florence ; Bonino (President of the Italian Hom. Institute), of Turin ; Cigliano, of Naples ; Fagiani, of Genoa.

*Portugal.*—Augusto de Mello and Daniel Tavares, of Lisbon.

*Switzerland.*—Batault, of Geneva ; Beck, of Monthey, Schædler, and Siégrist, of Basle.

*Russia.*—De Brasol, of St. Petersburg.

*America.*—Clark and Miss I. M. Rankine, of New York ; M. Wright, of Buffalo ; and Church, of Boston.

*Australia.*—Fisher, of Sydney.

Also, the Count Barbo and Madame the Duchess de Melzi d'Eril, of Milan, to whom we owe gratitude, as their collaboration is a striking proof of their attachment to homœopathic doctrine.

The characteristic note of the Congress of 1889 is the discussion on the employment of mixed medicines. It was the first time that such a proposal was brought to an important assembly. It was, as might be expected, unfavorably received. During the Congress in London, 1881, the employment of alternated medicines was discussed, when the subject was only skimmed. The present Congress devoted all of one session to discussing alternations and mixtures, which permitted the question to be carried to its true ground. Without doubt the future Congresses will continue anew the same subject, which will not be exhausted for some time to come. The Congress has recorded its disapprobation of Electro-Homœopathy. Their disavowal is aimed not so much at the employment of mixed medicines, but at the secret, so long guarded, as to the composition of these so-called mixtures, and all the procedures of Count Mattei that are incompatible with honesty in medicine, and, above all, completely foreign to the precise method of Hahnemann. This decision of the Congress of 1889 ought not to be compared with the famous condemnation of Antimony by the Parliament of Paris. What it has declared at present is that Electro-Homœopathy has nothing in common with Hahnemann's doctrine; that it carries a deceptive title, and that all homœopathic doctors refuse any connection with it.

(*Bibliothéque Homœopathique*, Sept., 1889.)

---

